# Editorial: Growth and pubertal development in paediatric endocrinology: from physiological pathways to clinical challenges

**DOI:** 10.3389/fped.2026.2020433

**Published:** 2026-10-20

**Authors:** Egidio Candela, Stefano Zucchini, Federico Baronio

**Affiliations:** 1 Pediatric Unit, IRCCS Azienda Ospedaliero-Universitaria di Bologna, Bologna, Italy; 2 Department of Medical and Surgical Sciences, Alma Mater Studiorum – University of Bologna, Bologna, Italy; 3 Diabetes and Endocrinology Clinic, Pediatric Unit, University Hospital, Arcispedale Sant’Anna, Ferrara, Italy

**Keywords:** central precocious puberty, growth hormone deficiency, IGF-1, microbiome, p450 oxidoreductase deficiency, puberty timing

The studies on statural growth and pubertal development have always represented the hallmark of pediatric endocrinology and a consistent number of studies has been published in the last 50 years. Although the knowledges on growth and, puberty have greatly improved, many questions remain unanswered, particularly about human-environment interactions, genetic background, neuroendocrine regulation, and nutritional and metabolic status. Our special issue, published jointly in *Frontiers in Endocrinology* and *Frontiers in Pediatrics*, emphasizes these aspects and aims to add new insights to the existing body of knowledge.

Early activation of the hypothalamic-pituitary-gonadal (HPG) axis, which underlies central precocious puberty (CPP), is one of the clearest examples of the complexity of these processes, and several colleagues have contributed to this topic.

In their study, Liu et al. analyzed polymorphisms in the estrogen receptor *β* (ER*β*) gene in girls with idiopathic CPP. Certain polymorphisms, primarily the ER*β* gene RsaI and Rraa haplotypes, appear to be associated with an increased risk of idiopathic CPP and may therefore serve as prognostic and risk factors.

Another study by Morrou et al. highlighted differential expression of circulating microRNAs between girls with CPP and healthy controls, with higher expression in the former of pathways associated with this neurological condition. Specifically, miR-148a-3p increased markedly in extracellular vesicles, supporting the hypothesis that post-transcriptional regulation may contribute to early activation of the HPG axis. The study also lays the groundwork for linking mechanistic research to the identification of minimally invasive biomarkers.

As mentioned earlier, environmental factors can also play a key role in this context.

Nie et al. focus specifically on the role of exposure to bisphenol A, widely used in plastic manufacturing, in precocious puberty, using computational analyses that link this substance to various potential molecular targets.

On the same topic, the mini-review by Amodeo et al. attempts to bring together the various causes, primarily genetic and environmental, underlying PPC in males. One period when environmental pressure was greatest was the COVID-19 pandemic, during which many centers, including ours, reported a significant increase in CPP cases (1, 2).

The increasing biological complexity of CPP has direct implications for its diagnosis. A particularly interesting study by Yuan et al., involving 1,077 premenarcheal girls with idiopathic CPP, found that BMI correlated with basal LH levels and other markers of pubertal development. These findings align with other global evidence, particularly because they highlight the need to assess patients beyond biochemical values and consider a broader range of factors (3). This issue is especially relevant to using basal LH as a diagnostic marker of HPG-axis activation (4).

The Endocrine Society's recent 2026 guidelines also point in this direction, suggesting an integrated approach that is not limited to biochemistry (5).

During puberty, there is also a particular window of opportunity to assess adrenal function, as shown in the study by Zhang et al., which used liquid chromatography coupled with tandem mass spectrometry in adolescent girls with polycystic ovary syndrome. These methods documented multiple alterations in androgen metabolic pathways in these patients, with the free androgen index also proving particularly effective for diagnosis, in line with other studies (6).

The transition from static categories toward longitudinal phenotypes is also evident in growth assessment. Gürlek et al. examined familial, constitutional, and combined forms of idiopathic short stature, emphasizing their heterogeneous developmental trajectories and the importance of pubertal timing in interpreting longitudinal growth in a retrospective cohort of 171 children. In particular, the study recommends that clinicians always conduct a longitudinal growth assessment before starting r-GH therapy, considering a wide range of parameters and the high rate of spontaneous improvement, especially after puberty. Although recombinant GH has an overall favorable safety profile, its systemic growth-promoting effects warrant appropriate clinical surveillance (7). Concerns have been raised about its potential influence on pre-existing structural abnormalities, such as Chiari malformation, although available evidence remains reassuring (8).

Ruiz del Olmo-Izuzquiza et al. prospectively assessed cardiac morphology and function alongside aerobic performance. Given the expected increase in left ventricular mass, the study observed physiological cardiac remodeling rather than pathological hypertrophy, with preserved systolic function and early subclinical improvements in cardiac contractility.

Alongside these safety considerations, one of the most promising and innovative tools to emerge in recent years is the use of predictive models to tailor follow-up for these patients. Ly et al., using interpretable predictive models in boys receiving growth hormone therapy, identified current IGF-1 SDS as the strongest predictor of subsequent IGF-1 levels, while treatment- and puberty-related variables provided additional predictive information.

A review by Li and Wang analyzed the effects of systemic glucocorticoid exposure during childhood, focusing on changes in myocardial structure and cardiac function. The data, broken down by age group, are still limited and suggest that structural changes most often manifest as treatment-reversible myocardial hypertrophy, whilst evidence supporting permanent structural damage remains insufficient.

At the opposite end of the spectrum, rare disorders of steroidogenesis remind us of the diagnostic value of integrating endocrine phenotyping with molecular genetics. Zhang et al. reported a patient with P450 oxidoreductase deficiency (PORD) carrying a previously unreported POR variant and complemented the case with a systematic review of 167 reported patients.

These studies certainly merit further investigation and larger validation samples; however, we are confident that they can provide new evidence and valuable food for thought for endocrinologists who will face the challenges of the future.
